# CRISPR interference-based gene repression in the plant growth promoter *Paenibacillus sonchi* genomovar Riograndensis SBR5

**DOI:** 10.1007/s00253-020-10571-6

**Published:** 2020-04-09

**Authors:** Luciana F. Brito, Kerstin Schultenkämper, Luciane M. P. Passaglia, Volker F. Wendisch

**Affiliations:** 1 Genetics of Prokaryotes, Faculty of Biology and CeBiTec, Bielefeld University, Bielefeld, Germany; 2 Department of Biotechnology and Food Science, NTNU, Norwegian University of Science and Technology, Trondheim, Norway; 3 Department of Genetics UFRGS, Universidade Federal do Rio Grande do Sul, Porto Alegre, Brazil

**Keywords:** *Paenibacillus sonchi*, CRISPR interference, dCas9, Sorbitol catabolism, Sporulation, Biofilms

## Abstract

Gene repression using the endonucleolytically deactivated dCas9 protein and sgRNAs (CRISPR interference or CRISPRi) is a useful approach to study gene functions. Here, we established CRISPRi in *Paenibacillus sonchi* genomovar Riograndensis SBR5, a plant growth promoting bacterium. CRISPRi system with sgRNAs targeting SBR5 endogenous genes *spo0A*, *yaaT* and *ydjJ* and plasmid-borne *gfpUV* was constructed and analyzed. Flow cytometry analysis revealed a significant decrease of reporter protein GFPUV signal in *P. sonchi* strains expressing *gfpUV* sgRNA in comparison with non-targeting controls. CRISPRi-based repression of chromosomal genes for regulation of sporulation *spo0A* and *yaaT* decreased sporulation and increased biofilm formation in SBR5. Repression of the sorbitol catabolic gene *ydjJ* revealed decreased specific activity of YdjJ in crude cell extracts and reduced biomass formation from sorbitol in growth experiments. Our work on CRISPRi-based gene repression serves as basis for gene function studies of the plant growth promoter *P. sonchi* SBR5. To our knowledge, the present study presents the first tool for gene repression established in *Paenibacillus* species.

**Table:** 

Key points • *CRISPRi toward gene repression was applied for the first time in Paenibacillus.* • *CRISPRi of spo0A and yaaT depleted spores and increased biofilms in SBR5.* • *CRISPRi-based ydjJ repression decreased specific activity of sorbitol dehydrogenase.*

Key points

• *CRISPRi toward gene repression was applied for the first time in Paenibacillus.*

• *CRISPRi of spo0A and yaaT depleted spores and increased biofilms in SBR5.*

• *CRISPRi-based ydjJ repression decreased specific activity of sorbitol dehydrogenase.*

## Introduction

CRISPR (clustered regularly interspaced short palindromic repeats) associated with the type II protein Cas9 is a well-known and well-established tool applied for genome editing of diverse organisms. Furthermore, the endonucleolytically deactivated mutant of Cas9 protein, named dCas9, still maintains sgRNA-guided DNA binding, which can interfere with RNA polymerase (Qi et al. 2013). Therefore, dCas9-based CRISPR interference (CRISPRi) has been applied for gene repression (Xu and Qi 2019).

CRISPRi-based gene repression has been applied in bacteria for many purposes, for instance, to redirect metabolic fluxes toward industrially relevant products (Cleto et al. 2016; Kim et al. 2017; Schultenkämper et al. 2020). Moreover, multiplexed CRISPRi was established in *Bacillus licheniformis* by targeting genes involved in byproduct synthesis and l-valine degradation pathway, resulting in up to 80% increase in l-valine titers (Zhan et al. 2020). In *Bacillus subtilis*, CRISPRi-mediated repression of 16 genes on the branch metabolic pathways of amino acid biosynthesis resulted in up to 0.75 g L^−1^ surfactin production (Wang et al. 2019). Recently, CRISPRi was established in the methylotrophic *Bacillus methanolicus*, controlling its sporulation process by repression of sporulation regulator Spo0A and controlling its mannitol catabolism by CRISPRi of mannitol-1-phosphate dehydrogenase *mtlD* gene (Schultenkämper et al. 2019).

*Paenibacillus riograndensis* (Beneduzi et al. 2010), that was recently reclassified as a genomovar of *P. sonchi* (Sant'Anna et al. 2017), is a Gram-positive, biotin auxotrophic, spore-forming bacterium. The strain SBR5 was isolated from wheat (*Triticum aestivum*) cultivated in fields in the south of Brazil (Beneduzi et al. 2008). This organism showed plant growth promoting characteristics, such as the ability to fix nitrogen (Fernandes et al. 2014) and to produce siderophores and indole-3-acetic acid (Beneduzi et al. 2010), and for pathogen biocontrol (Bach et al. 2016). The inoculation with SBR5 improved growth of wheat under greenhouse conditions (Campos et al. 2015). The complete genome sequence of *P. sonchi* genomovar Riograndensis SBR5 was determined, consisting of a single chromosome of 7,893,056 base pairs containing 6705 protein coding genes (Brito et al. 2015). Moreover, comprehensive transcriptome analyses enabled the characterization of its global transcriptional landscape (Brito et al. 2017a). The genome sequence of SBR5 has been used as basis for characterization of its nitrogen fixation system through differential RNA sequencing, revealing two *nif* gene clusters and one alternative nitrogenase *anf* gene cluster (Fernandes et al. 2014). Further, genome-based RNA sequencing under iron depletion showed expression of genes related to iron uptake and revealed signs of stress resistance displayed by increased expression of genes involved in siderophore transportation, sporulation, and DNA protection (Sperb et al. 2016).

Notwithstanding its great potential for application as plant growth promoting bacterium, *P. sonchi* genomovar Riograndensis SBR5 is still not characterized in depth. For characterization, the uses of genetic tools for gene expression and repression are advantageous in order to better understand a given process. Genetic tools for gene expression have been developed for *P. sonchi* SBR5, with a theta-replicating plasmid and a rolling circle-replicating plasmid operating in fluorescent reporter gene expression and generation of a biotin prototrophic strain (Brito et al. 2017b). However, up to now, gene knockout or repression tools have not yet been developed for this bacterium. Interestingly, the genetic tools for gene expression in *B. methanolicus* (Irla et al. 2016) were transferable to *P. sonchi* SBR5 (Brito et al. 2017b). Thus, CRISPRi-mediated gene repression tools developed for *B. methanolicus* (Schultenkämper et al. 2019) were chosen here as a starting point for the establishment of gene repression in SBR5.

While CRISPRi has been developed and is intensively used for model bacteria, there is a need to transfer CRISPRi technology for application in non-model bacteria. Therefore, we established CRISPRi in *P. sonchi* genomovar Riograndensis SBR5. As proof of concept, CRISPRi was used to repress genes controlling sporulation and biofilm formation in SBR5, besides a gene involved in sorbitol catabolism. The present study represents a valuable example of CRISPRi-based gene repression in the plant growth promoter *P. sonchi* SBR5 and serves as groundwork for future characterization studies.

## Material and methods

### Strains, plasmids, and oligonucleotides

All bacterial strains, plasmids, and oligonucleotides used in this study are listed in Table 1. *Paenibacillus sonchi* genomovar Riograndensis SBR5 (DSM 28159) was used as the expression host; *Escherichia coli* strain DH5α (Stratagene) was used as general cloning host.

**Table 1:** Description of strains, plasmids, and oligonucleotides of the present study

Strain	Characteristics	Origin
*Paenibacillus sonchi* genomovar Riograndensis SBR5	Wild-type strain, expression host	Beneduzi et al. (2010)
*Escherichia coli* DH5α	General cloning host	Stratagene
Plasmid	Characteristics	Origin
pNW33N	pNW33N derivative in which the *knt*-resistance gene was inserted, CmR, KanR	Irla et al. (2016)
piCas	pNW33N-derived plasmid carrying *dcas9*, driven by the mannitol-inducible m2p promoter, CmR, KanR	Schultenkämper et al. (2019)
piCas-*tspo0A*	piCas plasmid carrying the t*spo0A* sgRNA, targeting the non-template strand of *spo0A*, CmR, KanR	This study
piCas-*tyaaT*	piCas plasmid carrying the t*yaaT* sgRNA, targeting the non-template strand of *yaaT*, CmR, KanR	This study
piCas-*tydjJ*	piCas plasmid carrying the t*ydjJ* sgRNA, targeting the non-template strand of *ydjJ*, CmR, KanR	This study
piCas-*tgfpUV*	piCas plasmid carrying the t*gfpUV* sgRNA, targeting the non-template strand of *gfpUV*, CmR, KanR	This study
pBV2mp-*gfpUV*	pBVmp derivative for *gfpUV* expression under control of mdh promoter, AmpR, KanR	Irla et al. (2016)
Oligonucleotides annealed and inserted in piCas *Xba*I and *Ava*I site	Sequence* (5′-3′)	
*spo0A* fw	gggatataaacgttttatgataaatatCAACGCCATCTCGTGTAGAG	
*spo0A* rv	taacttgctatttctagctctaaaacCTCTACACGAGATGGCGTTG	
*yaaT* fw	gggatataaacgttttatgataaatatCTGTCTCCACAATTACACAC	
*yaaT* rv	taacttgctatttctagctctaaaacGTGTGTAATTGTGGAGACAG	
*ydjJ* fw	gggatataaacgttttatgataaatatATCAGGCAGCCGTGGTAGGG	
*ydjJ* rv	taacttgctatttctagctctaaaacCCCTACCACGGCTGCCTGAT	
*gfpUV* fw	gggatataaacgttttatgataaatatCATCTAATTCAACAAGAATT	
*gfpUV* rv	taacttgctatttctagctctaaaacAATTCTTGTTGAATTAGATG	

*Letters in lowercase represent plasmid overlaps

### Media and cultivation conditions

*E. coli* strains were routinely cultivated at 37 °C in lysogeny broth (LB; Bertani 1951) or on LB agar plates (1% w/v) supplemented with antibiotics (chloramphenicol 25 μg mL^−1^, kanamycin 50 μg mL^−1^) when needed.

In all experiments, *P. sonchi* strains were cultured in 500-mL shaking flasks containing 50 mL of CASO broth (DMSZ 220) or in 24-square deep well plates (Duetz et al. 2000) containing 3 mL of PbMM (*Paenibacillus* minimal medium; Brito et al. 2017a), shaking at 120 rpm and at temperature of 30 °C. For each condition tested, six biological replicates were used: three replications of bacterial cells were harvested for total RNA isolation, and three for further determination of growth characteristics. The optical density (OD) at 600 nm of the cultivated cells was measured throughout growth. Recombinant *P. sonchi* strains were routinely plated on 1% agar (w/v) CASO broth. When appropriate, culture media were supplemented with 2.5 μg mL^−1^ chloramphenicol and 50 mM mannitol as *dcas9* expression inducer. Glucose (100 mM) was used as standard carbon source for cultivation of *P. sonchi* in minimal medium, but 98.9 mM sorbitol was used as additional carbon source when appropriate.

### Plasmid construction and preparation of recombinant strains

**Fig. 1:**
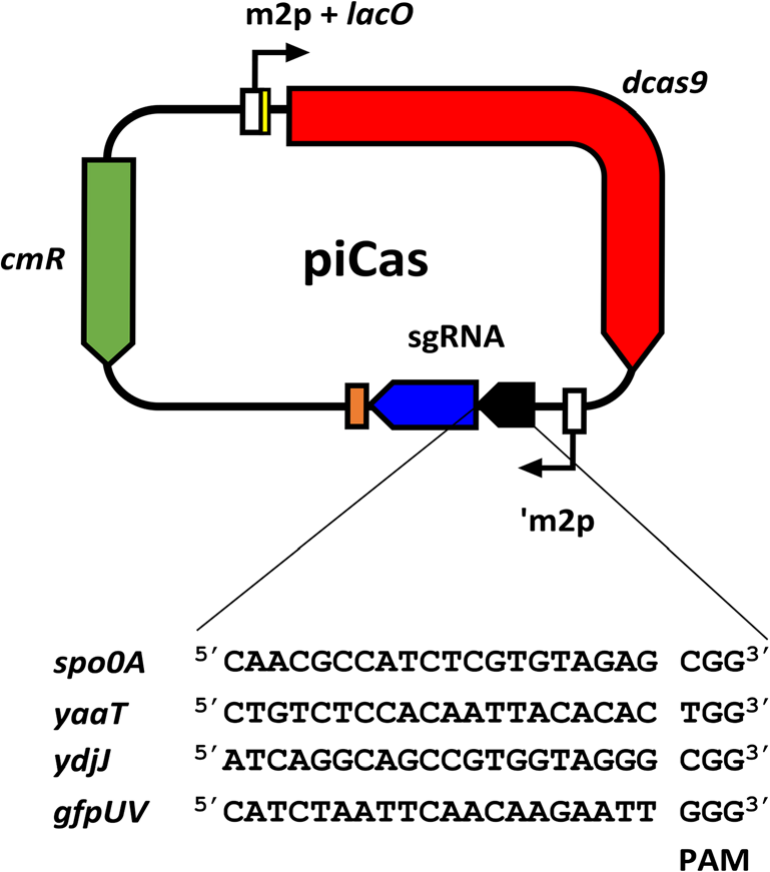
Schematic representation of plasmid piCas and sgRNA sequences used in this study. Vector pNW33N was used as backbone for contruction of piCas. *Bacillus methanolicus* m2p promoter has *lacI* operator sequence (*lacO;*yellow) inserted between the transcriptional start site and the *dcas9* coding sequence (red) to avoid expression in the cloning host *E. coli* (Schultenkämper et al. 2019). sgRNA transcription is driven by m2p promoter lacking its 5′UTR (′m2p). *Xba*I and *Ava*I sites are positioned upstream dCas9 handle (blue) and *Streptococcus pyogenes* terminator (orange) sequence (Schultenkämper et al. 2019). Those sites were used to linearize piCas vector for insertion of gene targeting sequence (black). The PAM regions and spacer sequences were selected from SBR5 genome sequence using the CRISPy-web tool (Blin et al. 2016)

Molecular cloning was performed as described by Sambrook and Russel (2001). Chemically competent cells of *E. coli* DH5α were prepared for cloning (Hanahan 1983). All the information about plasmid construction and oligonucleotide sequences are described in Table 1. The NucleoSpin^®^ Gel and PCR clean-up kit (Machery-Nagel, Düren, Germany) were used for PCR clean-up, and plasmids were isolated using the GeneJET plasmid miniprep kit (Thermo Fisher Scientific, Waltham, USA). The pNW33N derivative plasmid piCas (Schultenkämper et al. 2019) was double digested with *Xba*I and *Ava*I restriction enzymes (New England Biolabs, Ipswich, USA). Because CRISPRi targeting template DNA strand shows little repression effect (Qi et al. 2013; Cleto et al. 2016), we designed sgRNAs targeting non-template strands of *P. sonchi* genes. sgRNAs were selected by using CRISPy-web tool (Blin et al. 2016) and introduced to piCas plasmid *Xba*I and *Ava*I cloning site (Fig. 1). In order to build sgRNAs, oligonucleotides (Table 1) were annealed at 95 °C for 5 min and cooled to room temperature for 1 h. Later, their overlapping regions were joined by Gibson assembly (Gibson et al. 2009). For colony PCR, Taq polymerase (New England Biolabs) was used. The correctness of inserted DNA sequences was confirmed by sequencing in the sequencing facility of the Center for Biotechnology (University of Bielefeld, Germany). The constructed plasmids were transformed to *P. sonchi* SBR5 by means of magnesium-aminoclay method, as described by Brito et al. (2017b).

### Quantification of sporulation

*Paenibacillus sonchi* strains were cultivated in CASO broth as described above. After 6 h, when the cells were in exponential growth phase, the cells were transferred to fresh CASO broth containing sporulation salts (15 mg L^−1^ MnCl_2_, 1 g L^−1^ MgCl_2_, and 0.8 g L^−1^ CaCl_2_; Malvar et al. 1994). The endospores, middle stage spores, and non-spore cells were counted in technical triplicates. More than 100 cells were counted with a hemocytometer per condition and sample. If needed, suitable dilutions were used. The cells were examined by bright field microscopy (Zeiss^®^ Axio Lab.A1).

### Colony phenotyping of recombinant *P. sonchi* strains

Iris software, an image processing software that automatically quantifies several features of microbial colonies in high throughput (Kritikos et al. 2017), was used to determine the colony phenotype of *P. sonchi* strains. Therefore, the recombinant *P. sonchi* strains were cultivated in 50-mL CASO broth until reaching exponential phase (OD = 0.6), and 24 drops were stamped with a replicator on CASO agar plates containing or not mannitol for *dcas9* gene expression. Plates were imaged and analyzed with the IRIS software, in which colony opacity and size were determined.

### Quantification of biofilms

Biofilm formation was determined according to O'Toole (2011) microtiter dish biofilm formation assay. Recombinant *P. sonchi* strains were inoculated in round-bottomed microtiter plates (Greiner Cellstar^®^, 96-well cell culture plate) to an OD of 1, containing 200 μL CASO broth supplemented with expression inducer mannitol, when needed, in each well. The plates were incubated for 2 days in a 30 °C incubator. After incubation, the cells were dumped out by turning the microtiter plate over. Then, the plate was gently submerged in water. After shaking out the water, 200 μL of a 0.1% (w/v) crystal violet solution was added in each well. The microtiter plate was incubated at room temperature for 15 min. The solution was then rinsed out by washing the plate gently in water for several times, and the plate was turned upside down and dried overnight. To quantify the biofilm formation, 200 μL of 30% acetic acid was added to the wells to solubilize the crystal violet. The plate was incubated at room temperature for 15 min, and 200 μL of the solubilized solutions was transferred to a new flat-bottomed microtiter dish (Thermo Fisher Scientific, 96 wells). The absorbance was measured at 560 nm in a plate reader.

### GFPUV fluorescence measurement by flow cytometry

In order to quantify green fluorescent protein (GFPUV) mean fluorescence intensities (MFI) of *P. sonchi* SBR5 pBV2mp-*gfpUV* strains harboring CRISPRi plasmids and proper controls, cell cultures were analyzed by fluorescence activated cell scanning (FACS). Therefore, *P. sonchi* cells were cultured until reaching exponential phase and centrifuged for 10 min at 4000 rpm (5810R centrifuge, Eppendorf, Hamburg, Germany). The pellets were washed two times in NaCl 0.89%, and the OD of the cultures was adjusted to 0.5. The fluorescence of the cell suspension was measured using flow cytometer (Beckman Coulter, Brea, USA) and the data analyzed in the Beckman Coulter Kaluza® Flow Analysis Software. As described previously, the settings for the emission signal and filters within the flow cytometer for detection of GFPUV fluorescence were based on a 550/525 bandpass FL9 filter (Brito et al. 2017b).

### RNA isolation and qRT-PCR

For total RNA isolation, *P. sonchi* strains were cultivated in CASO broth containing 100 mM sorbitol and 50 mM mannitol to exponential phase (OD = 1). The cultivation was carried out in biological triplicates. Bacterial cells were harvested by 15 min centrifugation at 4 °C and 4000 rpm (5810R centrifuge, Eppendorf). The supernatant was discarded, and the pellets were immediately frozen in liquid nitrogen and stored at − 80 °C. In order to isolate RNA, the cell pellets were thawed in ice and the samples were homogenized by resuspending the cells in 100 μL TE buffer (10 mM Tris-HCl, 1 mM EDTA; pH 8) containing 5 mg mL^−1^ lysozyme. After 30 min incubation at 37 °C, total RNA was extracted using NucleoSpin® RNA kit (Macherey-Nagel) according to manufacturer’s instruction. Thereafter, RNA samples were treated with DNase digestion using RNase-free DNase Set and “RNeasy MinElute” kits (Qiagen, Hilden, Germany) to eliminate possible genomic DNA contamination. Furthermore, quality control with Taq polymerase (New England Biolabs) was performed in order to determine purity and integrity of isolated RNA. Additionally, total RNA concentration was measured using a spectrophotometer (NanoDrop^®^, ND-1000). Equal amounts of 50 ng of each RNA sample were used to perform quantitative real-time PCR (qRT-PCR). All qRT-PCRs were performed according to manufacturer’s instruction using the SensiFAST™ SYBR® No-ROX One-Step Kit (Bioline, London, UK) and the CFX96 cycler system (Bio-Rad, Hercules, USA). The temperature profile employed in all qRT-PCRs was 45 °C for 10 min (reverse transcription); 95 °C for 2 min; and 40 cycles of 95 °C for 5 s, 55 °C for 10 s, and 72 °C for 5 s; with melt curve analysis with measurements between 65 °C and 95 °C. The ΔCq method was used for calculations (Higuchi et al. 1992; Bustin et al. 2009). For each sample, three independent qRT-PCR experiments were performed.

### Determination of YdjJ specific activity

For determination of YdjJ activity, *P. sonchi* strains harboring the plasmids pNW33N, piCas or piCas-*tydjJ* strains were cultivated in flasks containing 50 mL CASO broth with 100 mM sorbitol and 50 mM mannitol, to circumvent growth deficits, until they reached an OD_600_ of approximately 1. Then, the cells were disrupted according to Brautaset et al. (2004). Crude extracts were used to determine YdiJ specific activity in an enzymatic assay using the Sorbitol Dehydrogenase Kit (MAK317, Sigma-Aldrich, St. Louis, USA). Sorbitol dehydrogenase activity was assayed according to manufacturer’s instructions at room temperature and 565 nm using a spectrophotometer (Shimadzu, UV-1800). One unit (U) of YdjJ catalyzed the conversion of 1 mol of d-sorbitol to fructose per minute at pH 8.2.

## Results

### CRISPRi-mediated repression of plasmid-borne GFPUV

In order to analyze CRISPRi efficiency in *P. sonchi* SBR5 cells, we chose to express reporter gene *gfpUV* sgRNA in SBR5 using the plasmid pBV2mp-*gfpUV*. sgRNA-guided dCas9 activity resulted in a significant decrease of GFPUV medium fluorescence intensity (MFI). GFPUV MFI in *P. sonchi* SBR5(pBV2mp-*gfpUV*) cells expressing dCas9 and *gfpUV* sgRNA was about 3 times lower than in the same strain expressing dCas9 alone (Table 2). Thus, repression of a highly expressed gene on a plasmid by CRISPRi could be demonstrated.

**Table 2:** CRISPRi-based repression of plasmid-borne *gfpUV* gene in *P. sonchi* SBR5. *Paenibacillus* sonchi SBR5(pBV2mp-gfpUV) carried *gfpUV* coding sequence targeted by sgRNA-guided dCas9. GfpUV mean fluorescence intensity (MFI) of recombinant cells was determined by means of flow cytometry. Data represent means and standard deviations of technical triplicates. Different numbers represent significant differences by Scott-Knott test (*p* < 0.05)

	GfpUV MFI			
SBR5(pBV2mp-*gfpUV*)(pNW33N)	1.49	±	0.33	(a2)
SBR5(pBV2mp-*gfpUV*)(piCas)	1.33	±	0.23	(a2)
SBR5(pBV2mp-*gfpUV*)(piCas-*tgfpUV*)	0.41	±	0.07	(a1)

### Effect of *ydjJ* gene repression on *P. sonchi* SBR5 grown in d-sorbitol

**Fig. 2:**
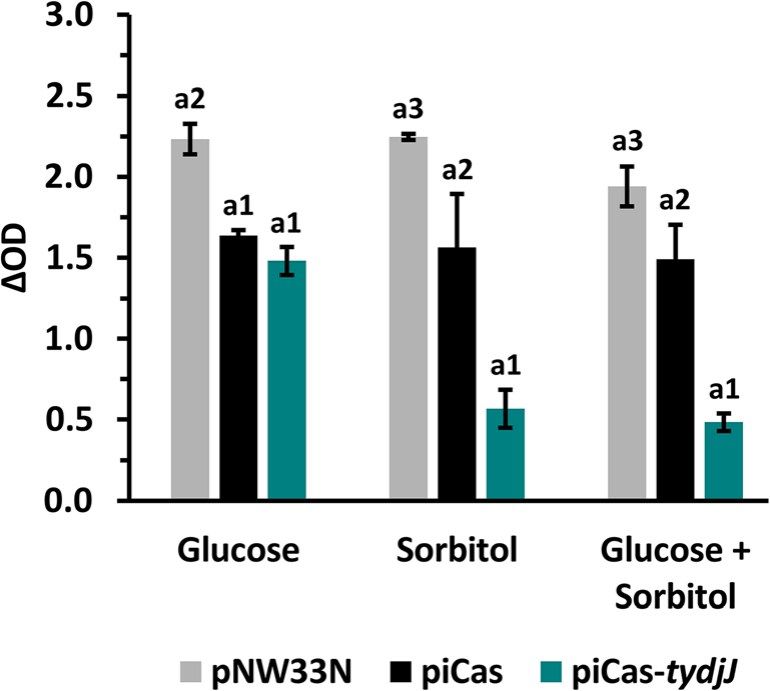
dCas9 targeting sorbitol dehydrogenase coding gene *ydjJ* reduced biomass formation of *P. sonchi* SBR5 in growth medium containing sorbitol. Final growth (ΔOD) of *P. sonchi* SBR5 cultivated in deep well plates (Duetz et al. 2000). SBR5 cells carried pNW33N, piCas9, or piCas9-*tydjJ* plasmids. Glucose (100 mM), sorbitol (98.9 mM) or glucose (50 mM), and sorbitol (49.45 mM) were used as sole or combined carbon sources in PbMM medium. ΔOD values represent the difference between the OD of cell cultures at the end of exponential phase and the OD at inoculation. The error bars represent standard deviation of technical triplicates. Different numbers represent significant differences by Scott-Knott test (*p* < 0.001)

After having shown that CRISPRi worked to repress a gene on a plasmid, we chose to repress a chromosomal gene. Based on the complete genome sequence of SBR5 (Brito et al. 2015), we selected a catabolic gene, namely the d-sorbitol dehydrogenase gene *ydjJ*, as CRISPRi target. *Paenibacillus sonchi* SBR5 carrying the plasmid piCas9-*tydjJ* and the strains carrying empty vector and non-targeting control plasmids were cultivated in minimal medium with d-sorbitol as sole carbon source and for comparison in glucose minimal medium. As expected, *P. sonchi* SBR5(pNW33N) was able to grow in glucose and d-sorbitol either as sole or as combined carbon sources (Fig. 2). However, expressing dCas9 alone in the non-targeting SBR5(piCas) strain surprisingly resulted in significantly decreased biomass formation in comparison with the empty vector control, regardless of the provided carbon source. Moreover, the biomass formation of SBR5(piCas-*tydjJ*) on glucose was compromised, but comparable with the non-targeting control (Fig. 2). In minimal media with d-sorbitol as sole or combined carbon source, SBR5 expressing *ydjJ* sgRNA resulted in 3 and 2 times less biomass formation in comparison with the empty vector control and non-targeting control, respectively (Fig. 2). This result indicated that YdjJ may play a crucial role in d-sorbitol catabolism in *P. sonchi* SBR5.

**Fig. 3:**
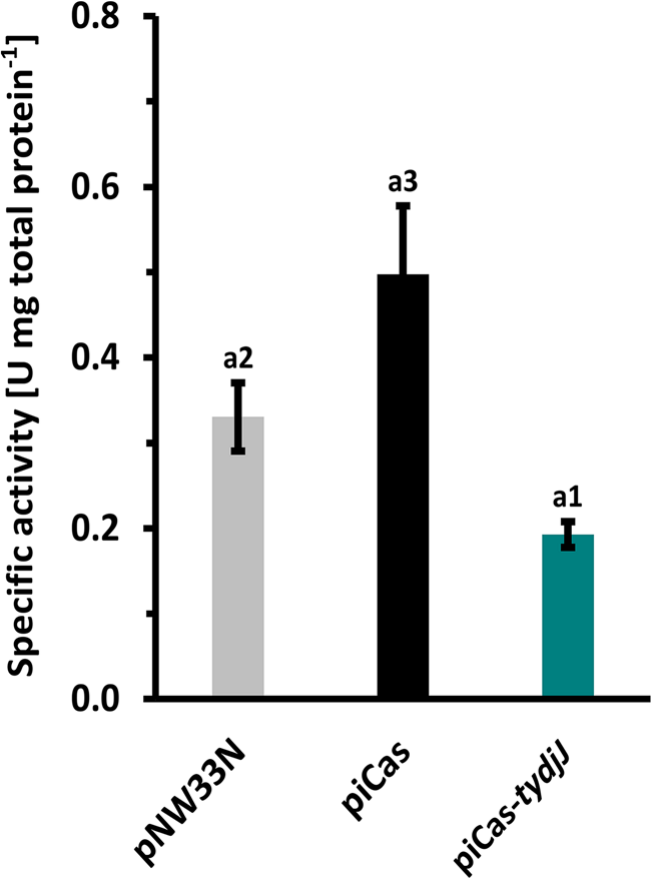
CRISPR interference-based gene repression decreased specific activity of sorbitol dehydrogenase YdjJ in crude extracts of *P. sonchi* SBR5. Specific activities (U mg total protein^−1^) of SBR5 strains harboring plasmids pNW33N, piCas, or piCas-*tydjJ*. One unit (U) of YdjJ catalyzed the conversion of 1 mol of d-sorbitol to fructose per minute at pH 8.2. Crude extracts were prepared after growth in CASO broth supplemented with 100 mM sorbitol and 50 mM mannitol. Error bars represent standard deviations of technical triplicates. Different numbers represent significant differences by Scott-Knott test (*p* < 0.05)

Pursuing more evidence of YdjJ CRISPRi in *P. sonchi* SBR5, we determined the YdjJ specific enzyme activity in crude extracts of SBR5(piCas-*tydjJ*) in comparison with proper controls. YdjJ specific activity in SBR5(piCas-*tydjJ*) was 0.2 ± 0.0 U mg total protein^−1^, thus less than half of the activity observed in the non-targeting control strain (0.5 ± 0.1 U mg total protein^−1^). Surprisingly, while the highest YdjJ specific activity was reached by the non-targeting SBR5(piCas) strain, the empty vector control strain SBR5(pNW33N) presented YdjJ specific activity of 0.3 ± 0.0 U mg total protein^−1^ (Fig. 3). Nevertheless, targeting *ydjJ* led to about 33% less enzyme activity in comparison with the empty vector control and this difference was statistically significant (*p* < 0.05; Fig. 3). Even though CRISPRi-based attenuation of *ydjJ* led to only partial depletion of YdjJ activity, these results illustrate that repression of a chromosomally encoded catabolic gene by CRISPRi could successfully be demonstrated, and the functional role of d-sorbitol dehydrogenase YdjJ for utilization of d-sorbitol could be revealed.

### CRISPRi targeting sporulation regulatory gene *spo0A* decreased its mRNA level

**Fig. 4:**
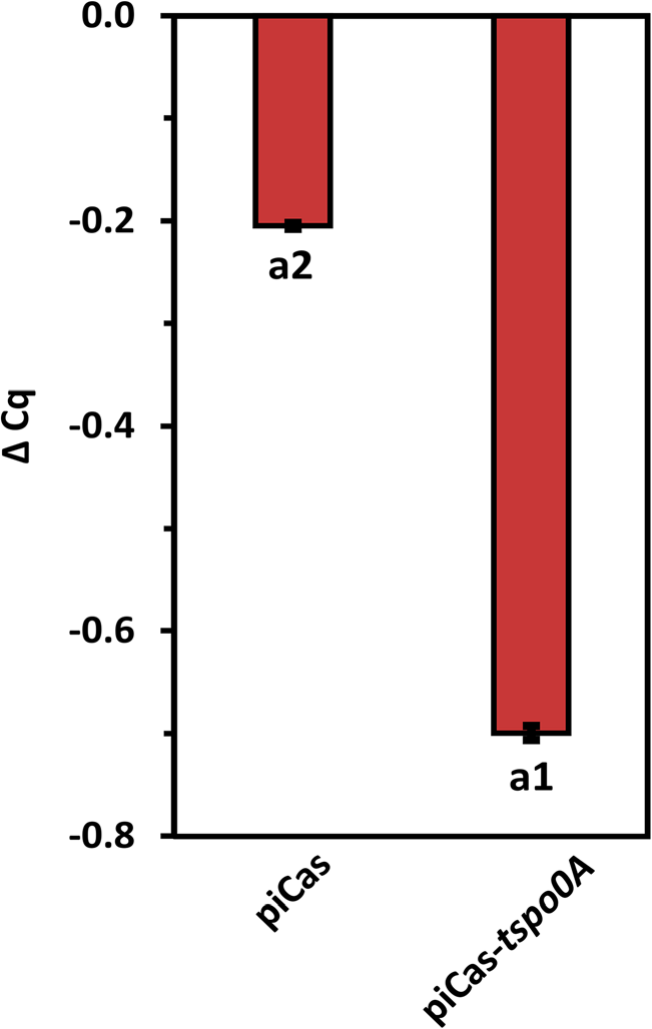
qRT-PCR revealed CRISPRi *spo0A* gene repression in *P. sonchi* SBR5. ΔCq expression of *spo0A* gene in the SBR5 strain piCas-*tspo0A* in comparison with non-targeting piCas control. Cq values obtained from *dcas9* gene were used as normalization factor. ΔCq expression means of biological triplicates are depicted, and error bars represent standard deviations of samples. Different numbers represent significant differences by Scott-Knott test (*p* < 0.05)

In order to test if CRISPRi can be applied to repress regulatory genes of *P. sonchi*, we chose to target the sporulation regulatory gene *spo0A*. The extent of gene repression of the recombinant *P. sonchi* SBR5(piCas-*tspo0A*) generated in the present study was quantified by qRT-PCR. The expression of SBR5 chromosomal gene *spo0A*, here targeted by sgRNA-guided dCas9, was quantified (Cq) and normalized by the Cq expression of *dcas9* gene (ΔCq). The relative qRT-PCR data of the knockdown strain revealed significantly reduced *spo0A* mRNA level (*p* < 0.05) in comparison with RNA samples from SBR5 control strain piCas (Fig. 4).

### Hemocytometer spore counts of *P. sonchi* cells targeting *spo0A* and *yaaT*

**Fig. 5:**
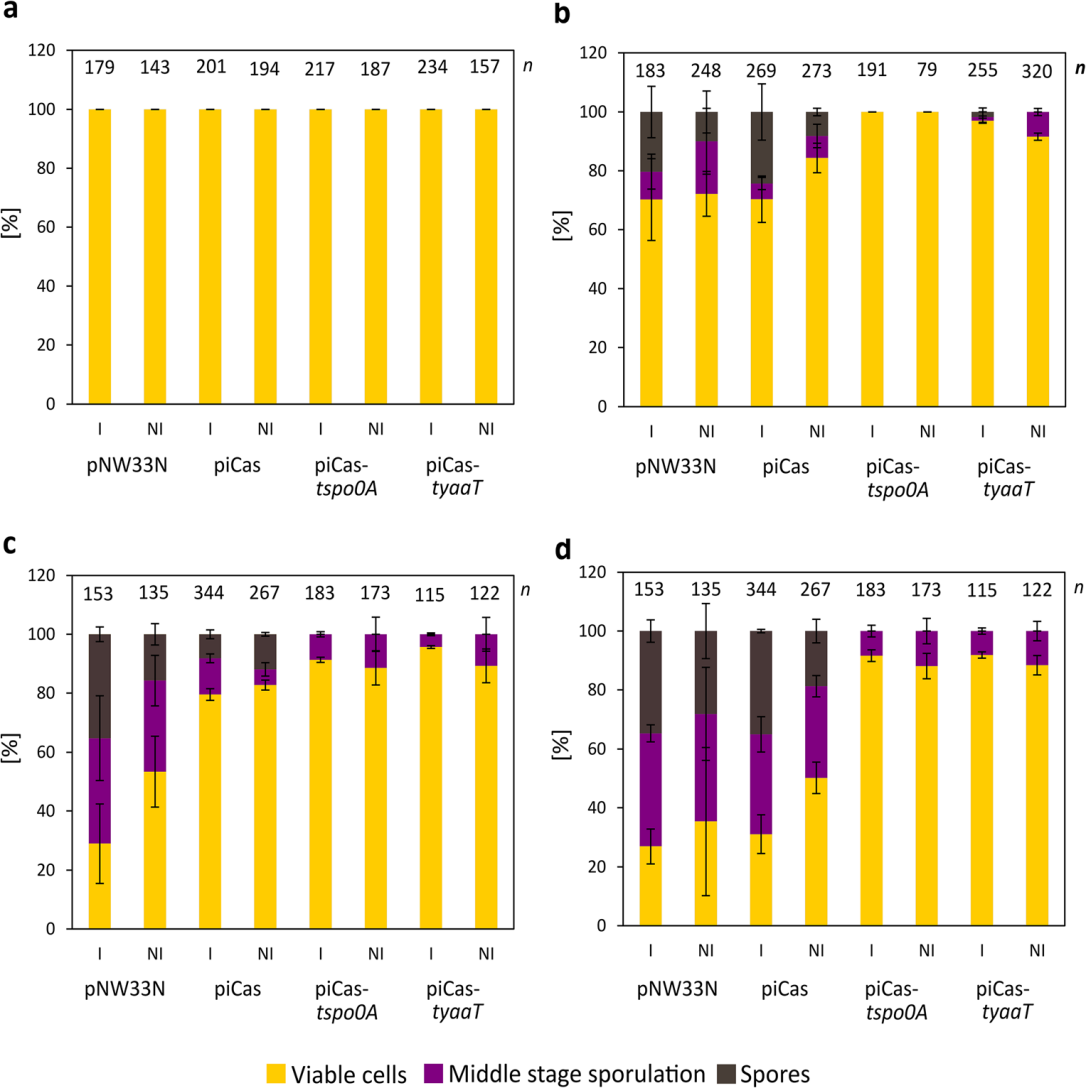
CRISPR interference of *spo0A* and *yaaT* genes reduced hemocytometer spore counts of *P. sonchi* SBR5 cells. SBR5 cells carrying plasmids pNW33N, piCas, piCas-*tspo0A*, or piCas-*tyaaT* were cultivated in CASO broth (DSMZ 220). Spores were counted by means of hemocytometer method at 0 (**a**), 24 (**b**), 48 (**c**), and 72 (**d**) hours after transfer to CASO broth containing sporulation salts (Malvar et al. 1994). CASO broth was supplemented (I) or not (NI) with 50 mM mannitol to induce *dcas9* expression. Percentage of viable cells, middle stage sporulation, and spores is given as means and standard deviations of technical triplicates

Next, we quantified the effect of CRISPRi on sporulation of *P. sonchi* SBR5 in media containing sporulation salts, by hemocytometer counts. In addition to targeting *spo0A*, *yaaT* was chosen as related regulatory sporulation gene. The percentages of viable SBR5 cells, cells in middle sporulation stage, and spores were calculated from the microscopic analysis. In the first quantification (*t* = 0, i.e., before transfer to medium with sporulation salts), SBR5 cells carrying the empty vector control plasmid pNW33N, the non-targeting piCas plasmid or piCas-*tspo0A* and piCas-*tyaaT* plasmids were all viable (Fig. 5a). Initial spore formation could be seen at 24 h of cultivation in CASO broth for SBR5 strains pNW33N (10% ± 7%) and piCas (8% ± 1%) (Fig. 5b). However, when the cultivation broth was supplemented with *dCas9* expression inducer mannitol, the percentage of spores slightly increased for SBR5 strains pNW33N and piCas (20% ± 9% and 24% ± 10% of spores from total cells, respectively) (Fig. 5b). Even though some spores were observed in mannitol-induced SBR5(piCas-*tyaaT*) at 24 h (2% ± 1%) (Fig. 5b), no spores were observed in the analyzed cells of the same strain in later time points (Fig. 5c and d). Cells in middle sporulation stage were observed at 24 h, counting 9% ± 6%, 5% ± 2%, and 1% ± 2% of induced pNW33N, piCas, and piCas-*tyaaT* cells, respectively (Fig. 5b). In contrast to SBR5 control strains pNW33N and piCas and the CRISPRi targeting strain piCas-*tyaaT*, SBR5(piCas-*tspo0A*) did not proceed to the middle stage of sporulation nor fully developed sporulation at 24 h that occurred independently of mannitol addition to the cultivation medium (Fig. 5b). Although CRISPRi repression of *spo0A* and *yaaT* genes resulted in total suppression of formed spores, SBR5(piCas-*tspo0A*) and SBR5(piCas-*tyaaT*) cultures, induced with mannitol or not, presented approximately 10% of cells in middle sporulation stage at 48 and 72 h (Fig. 5c and d). For the negative control strains pNW33N and piCas9, the percentage of cells in middle stage sporulation and formed spores increased dramatically in the late stationary phase, from 48 to 72 h (Fig. 5c and d). During this final stage of cultivation, sporulation increased further, as in the end 35% ± 4% and 35% ± 1% of the induced pNW33N, and piCas cells, respectively, were fully sporulated (Fig. 5d). Furthermore, non-induced cells of pNW33N and piCas presented approximately 20% sporulation (Fig. 5d). Hence, even though middle sporulation stage cells were observed along the growth, when dCas9 targeted the sporulation genes *spo0A* and *yaaT*, no formed spores were observed in up to 72 h (Fig. 5). Thus, targeting sporulation regulatory genes *spo0A* and *yaaT* slowed progress of sporulation dramatically (Fig. 5).

### Colony phenotyping analysis

**Fig. 6:**
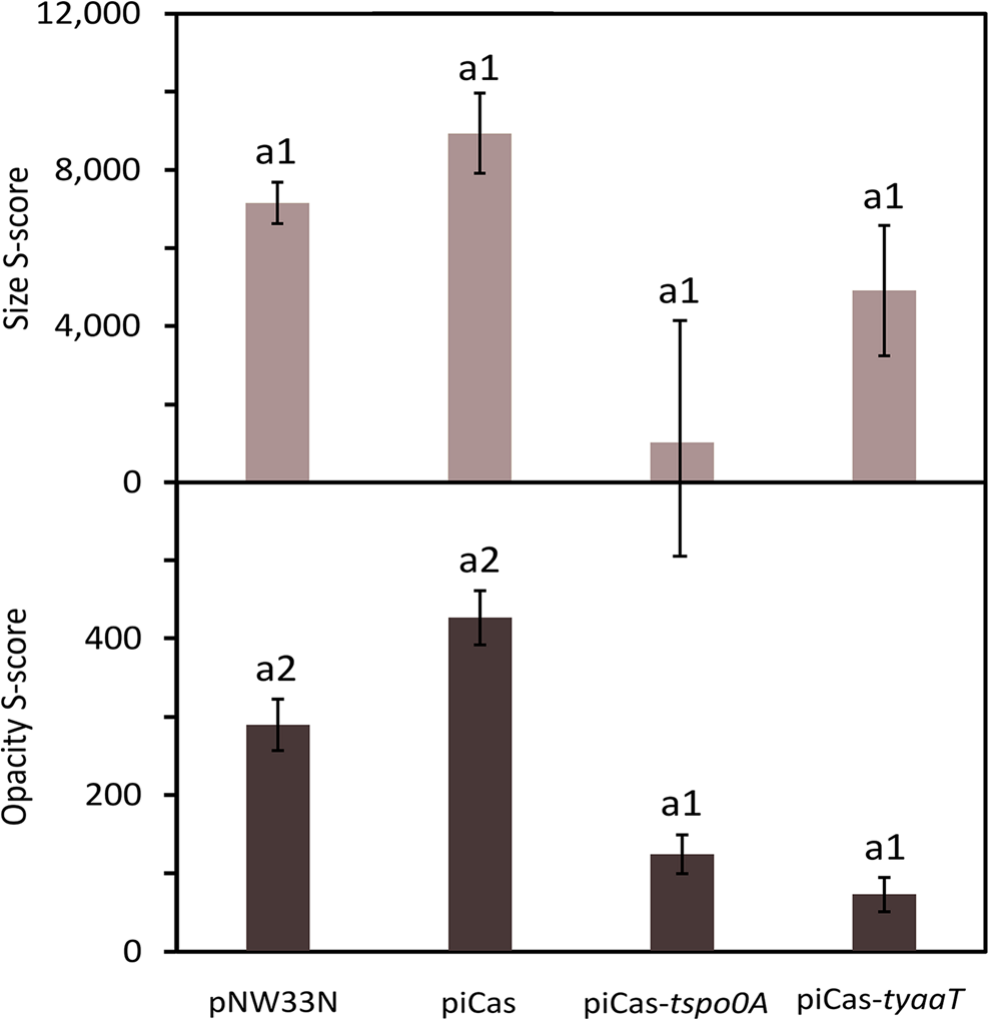
CRISPR interference of *spo0A* and *yaaT* genes altered colony phenotypes of *P. sonchi* SBR5. Equal volumes of SBR5 cell suspension carrying plasmids pNW33N, piCas, piCas-*tspo0A*, or piCas-*tyaaT* were transferred to agar CASO broth (DSMZ 220) plates. Plates were imaged for IRIS software analysis of colony size and opacity. Size S-score values represent the colony area in pixels; opacity S-scores represent the sum of the brightness values for all the pixels in the colony (Kritikos et al. 2017). High S-score values of opacity and colony size represent cell sporulation. Bars represent IRIS S-score means and error bars represent standard deviations of 24 equally spaced colonies. Different numbers represent significant differences by Scott-Knott test (*p* < 0.001)

CRISPRi targeting *spo0A* and *yaaT* genes in *P. sonchi* SBR5 resulted in strong reduction of spore formation (Fig. 5). To independently assess sporulation, we analyzed colony opacity since colonies of sporulating SBR5 present an opaque aspect in comparison with non-sporulating colonies. Therefore, colonies from *P. sonchi* SBR5 carrying the plasmids pNW33N, piCas, piCas-*tspo0A*, or piCas-*tyaaT* were imaged, and their size and opacity were scored using the IRIS software. The average colony size was similar for the sporulating colonies from SBR5(pNW33N) and SBR5(piCas) and the non-sporulating colonies from SBR5(piCas-*tspo0A*) and SBR5(piCas-*tyaaT*) (Fig. 6). Furthermore, while colonies of SBR5 expressing *spo0A* and *yaaT* sgRNAs remained translucent, sporulating SBR5(pNW33N) and SBR5(piCas) colonies presented high opacity scores (Fig. 6). Opacity score values for SBR5 strains carrying plasmids pNW33N and piCas were about 2-fold and 4-fold higher, respectively, in comparison with that in the strains expressing sgRNAs (Fig. 6). Taken together, targeting *spo0A* and *yaaT* revealed reduced sporulation not only by hemocytometer analysis of cells in liquid medium (Fig. 5), but also by colony opacity analysis of cells grown an agar plates (Fig. 6).

### Effect of CRISPRi on biofilm formation by *P. sonchi*

**Fig. 7:**
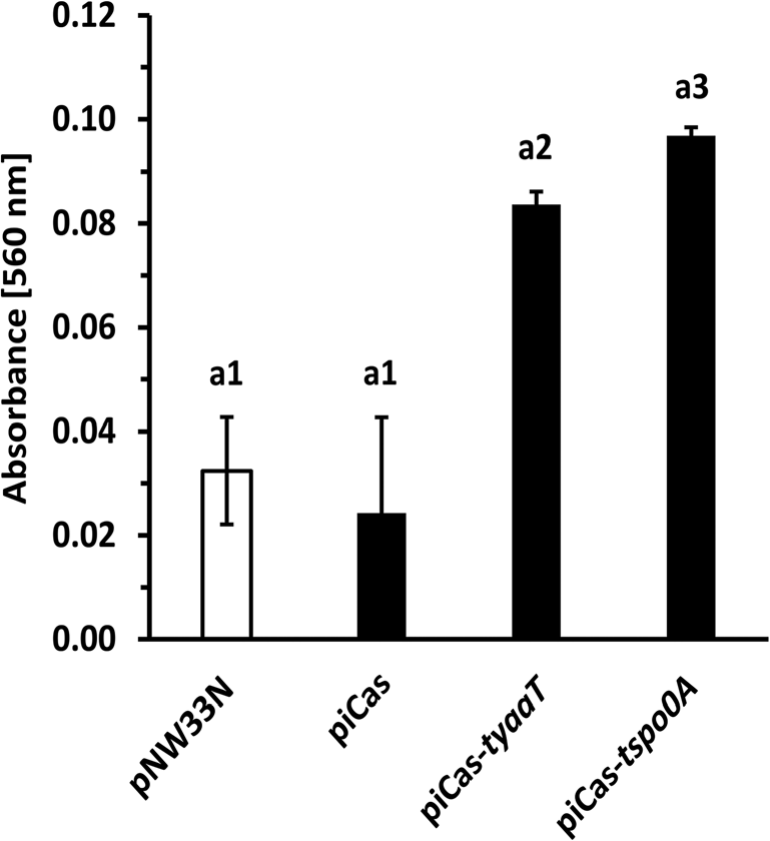
Crystal violet quantification revealed increased biofilm formation upon CRISPRi-mediated repression of *spo0A* and *yaaT* genes in *P. sonchi* SBR5. SBR5 cells carrying plasmids pNW33N, piCas, piCas-*tspo0A*, or piCas-*tyaaT* were cultivated in CASO broth (DSMZ 220) and transferred to 96 microwell plates. Three-day-old biofilms attached to the bottom of the individual wells in a 96-well plate were stained with crystal violet for absorbance readings (560 nm) derived from methanol elution of the crystal violet stain. Crystal violet absorbance means are depicted, and error bars represent standard deviation of 96 individual wells. Different numbers represent significant differences by Scott-Knott test (*p* < 0.001)

Spo0A regulates biofilm development in *B. subtilis* and *B. methanolicus* (Hamon and Lazazzera 2001; Bustin et al. 2009; Schultenkämper et al. 2019). To investigate biofilm formation in *P. sonchi* strains expressing *spo0A* and *yaaT* sgRNAs, biofilm formation was measured in a crystal violet assay. CRISPRi-based repression of both *spo0A* and *yaaT* genes led to approximately 3-fold increase in crystal violet signal (560 nm) in comparison with empty vector and non-targeting vector controls (Fig. 7). This result reflects the negative impact of Spo0A and YaaT on biofilm formation by SBR5. Thus, as expected, significant enhancements of biofilm formation were observed upon targeting *spo0A* and *yaaT*, although less dominant in the latter case (Fig. 7).

## Discussion

We developed CRISPRi of plasmid and chromosomal genes in the plant growth promoter *P. sonchi* SBR5 for the first time. Based on the CRISPRi-mediated gene repression system developed for the methylotrophic *B. methanolicus* (Schultenkämper et al. 2019), we showed repression of *gfpUV* expressed from a plasmid in a fluorescence assay. Evaluation of CRISPRi-based repression of fluorescent proteins is a reliable and often used method to test CRISPRi activity in bacteria. dCas9 and sgRNA targeting chromosome integrated EYFP (enhanced yellow fluorescent protein) gene conferred effective and stable EYFP suppression in *Synechococcus elongatus* (Huang et al. 2016). Moreover, targeting a genome-integrated gene coding for a red fluorescent protein (*rfp*) in *C. glutamicum* resulted in reduced RFP fluorescence (Cleto et al. 2016). In both cases, CRISPRi was highly efficient, with less than 1% remaining reporter protein signal. Similarly, targeting plasmid GFP reporter gene in *Pseudomonas putida* decreased fluorescence intensity to background levels (Kim et al. 2020). In this study, flow cytometry analysis revealed substantial drop of plasmid-borne GFPUV fluorescence (~ 70%) in *P. sonchi* SBR5 expressing dCas9 and *gfpUV* targeting sgRNA, but about 30% of the reporter signal remained (Table 2). Comparable results have been reported for *Klebsiella pneumoniae*, where plasmid-derived enhanced green fluorescent protein (EGFP) expression level was reduced to 10–15%, depending on the different targeted regions of promoter upstream of EGFP gene (Wang et al. 2018). Variations in CRISPRi activities might be due to the fact that CRISPRi regulation levels are ruled by sgRNA design, being highly dependent on PAM site variants and gene target locations (Tan et al. 2019). Even though our study did not compare the effect of various sgRNAs on *gfpUV* repression, the efficiency of the system could be confirmed by the repression of chromosomal genes, which resulted in 3-fold reduction of *spo0A* relative expression (Fig. 4) and clear phenotypic changes when genes *ydjJ*, *spo0A*, and *yaaT* were targeted.

CRISPRi of the chromosomally encoded *P. sonchi* gene coding for putative d-sorbitol dehydrogenase YdjJ revealed that a catabolic gene can be repressed by CRISPRi and corroborated its hypothetical function in sugar alcohol utilization. Upon targeting *ydjJ* biomass formation from d-sorbitol (Fig. 3) and NAD-dependent d-sorbitol dehydrogenase (EC 1.1.1.14) activity in crude extracts was reduced (Fig. 2). In the genome sequence of *P. sonchi*, *ydjJ* is annotated to code for NADP dependent alcohol dehydrogenase (Brito et al. 2015), and it shares 43.82% protein identity with d-sorbitol dehydrogenase from *B. subtilis* (Ng et al. 1992) and 42.94% with that enzyme from *Bacillus fructosus* (Uwajima 1999). The *B. subtilis* enzyme is active with other sugar alcohols, but possesses higher affinity for d-sorbitol (*K*_M_ = 11 mM, *V*_max_ = 6.25 μM min^−1^) than for other sugar alcohol substrates (Ng et al. 1992). Crude extracts of *B. fructosus*, efficient in producing d-fructose from d-sorbitol, reach a specific activity of d-sorbitol dehydrogenase of 0.0021 U mg total protein^−1^, yielding 100% of d-sorbitol conversion (Uwajima 1999). In our study, CRISPRi of *ydjJ* decreased YdjJ activity and biomass formation from d-sorbitol significantly, but not completely (Figs. 2 and 3). Incomplete repression of gene coding for catabolic proteins has been reported previously: CRISPRi of *B. methanolicus* mannitol-1-phosphate 5-dehydrogenase gene *mtlD* resulted in substantial, but not complete decrease of biomass formation from mannitol as well as of MtlD specific activity (Schultenkämper et al. 2019). As an alternative explanation, it is possible that the genome of *P. sonchi* encodes other proteins with side activity as d-sorbitol dehydrogenase. For example, xylitol dehydrogenase from *Gluconobacter oxydans* (EC 1.1.1.-) catalyzes the conversion of d-sorbitol to d-fructose (Liu et al. 2019). However, it remains to be studied if such enzymes exist in *P. sonchi* and/or whether *ydjJ* gene repression was incomplete.

It is known that the entry to sporulation in bacilli is closely regulated by a cascade of genetic events started by the transcriptional master regulator Spo0A (Tan and Ramamurthi 2014). Upon histidine kinase phosphotransfer, *B. subtilis* Spo0A initiates response processes such as biofilm formation, cannibalism, and sporulation (Hamon and Lazazzera 2001; Ellermeier et al. 2006; Veening et al. 2009). CRISPRi-mediated gene repression led to down regulation of *P. sonchi spo0A* (Fig. 4), which resulted in strong reduction of sporulated cells (Fig. 5). Likewise, CRISPRi system was successfully used to repress Spo0A in *B. methanolicus*, resulting in substantial decrease of sporulated cells (Schultenkämper et al. 2019). The decrease of sporulation ability could also be observed in *Clostridium*, when CRISPRi system was applied to target *spo0A* gene in *Clostridium beijerinckii* (Li et al. 2016). Moreover, colony opacity increases with the level of sporulation (White et al. 2006). Our colony phenotyping analysis confirmed that and showed high opacity scores of colonies from non-targeting control strains, whereas colonies from strains with sgRNA targeting *spo0A* gene remained translucent (Fig. 6). Thus, the crucial role of Spo0A in *P. sonchi* sporulation was confirmed. Furthermore, CRISPRi of *spo0A* impacted biofilm production of *P. sonchi* (Fig. 7). Previously, CRISPRi of quorum sensing gene *luxS* was used to inhibit biofilm formation in *E. coli* (Zuberi et al. 2017). Later, biofilm formation and swarming motility were strongly inhibited by CRISPRi of environmental sensor histidine kinase gene *gacS* in *Pseudomonas fluorescens* (Noirot-Gros et al. 2019). In *B. subtilis*, mutations in *spo0A* caused a defect in biofilm formation (Hamon and Lazazzera 2001). In contrast, targeting *spo0A* with CRISPRi increased formation of biofilms in *P. sonchi* (Fig. 7). As in the present study, Schultenkämper et al. (2019) reported increased biofilm formation of *B. methanolicus* when its *spo0A* gene was downregulated by means of CRISPRi. That might be explained by the fact that bacterial cells form biofilms in opposition to spores in face of low level of Spo0A phosphorylation (Hamon and Lazazzera 2001). Here, few spore counts were observed in *P. sonchi* cultures expressing *spo0A* sgRNA (Fig. 5), indicating some *spo0A* expression. Hence, a low level of *spo0A* expression in *P. sonchi* should increase its biofilm formation.

CRISPRi in *P. sonchi* showed that its sporulation process is driven by Spo0A protein and that targeting *spo0A* gene impacted biofilm formation. In addition, an extension of sporulation and biofilm regulation could be demonstrated. The *B. subtilis* protein YaaT interacts with the proteins YmcA and YlbF forming a stable ternary complex that acts upstream of Spo0A, accelerating its production (Carabetta et al. 2013). In accordance with the results obtained by downregulating *spo0A*, targeting *yaaT* alone with CRISPRi system resulted in a strong decrease of sporulation (Figs. 5 and 6) and an increase of biofilm formation (Fig. 7). These results are in accordance with the findings in *B. subtilis* in which YaaT directly influenced Spo0A (Carabetta et al. 2013; Hosoya et al. 2002).

Our study demonstrated CRISPRi employing type II Cas9 modified dCas9 protein as an efficient gene repression tool for *P. sonchi*. However, a toxic effect of dCas9 protein was observed in our experiments, for example, since growth in sorbitol/glucose of the non-targeting control strain SBR5(piCas) was significantly affected as compared with the control strain bearing the parental plasmid pNW33N lacking the *dcas9* gene (Fig. 2). It is widely observed that Cas9 and its mutant dCas9 may exhibit off-target activity and/or sequence-specific toxicity in target organisms such as *E. coli* (Cui et al. 2018; Hamilton et al. 2019) and clostridia (Xu et al. 2015). Previously, we observed cloning difficulties with plasmid piCas in *E. coli* DH5α which could be overcome by cloning of Lac repressor operator sequence (*lacO*) at the transcriptional starting site of the employed m2p promoter (Schultenkämper et al. 2019). In the target organism *P. sonchi*, the issue of toxicity might be circumvented by exchanging the promoter that drives the expression of *dcas9* gene. The m2p promoter used in the present study is functional and inducible by 40 mM mannitol in *P. sonchi* SBR5 (Brito et al. 2017b). Mannitol is not a gratuitous inducer since it is a carbon source utilized by *P. sonchi* SBR5 (Beneduzi et al. 2010). Moreover, the m2p promoter leads to significant background gene expression (Brito et al. 2017b). This leaky expression of dCas9 might be sufficient to cause cellular toxicity in *P. sonchi*. A tight regulation of *dcas9* is pivotal to overcome the background expression of this gene and consequently alleviate toxicity (Cho et al. 2018; Cui et al. 2018; Hofmann et al. 2019). dCas9 toxicity could also be mitigated in *E. coli* by mutating dCas9 PAM binding site and fusing it to TetR-family PhlF repressor in order to enhance DNA binding (Zhang and Voigt 2018). Moreover, the use of truncated sgRNA regions can decrease undesired Cas9 off-target double strand breaks (Fu et al. 2014). Altogether, our results of sporulation control and sorbitol catabolism control indicated strong on-target dCas9 repression. Thus, we conclude that despite the disadvantage of some levels of toxicity, the CRISPRi system established here is favorable for gene function studies in the plant growth promoting *P. sonchi* SBR5.

Our study opens doors for further gene function research in *P. sonchi* SBR5. More advanced tests may be amenable in the future including comparison of multiple sgRNAs pursuing higher CRISPRi efficiency levels and multiplexed repression allowing to target whole metabolic pathways rather than single genes. This has been shown in microbes in previous studies, e.g., an array of four sgRNAs was successfully applied for elimination of *n*-butanol byproducts in *E. coli* (Kim et al. 2017) and the simultaneous CRISPRi-mediated repression of seven genes competitive for β-amyrin synthesis in yeast (Ni et al. 2019). Furthermore, based on our demonstration of CRISPRi with dCas9 in *P. sonchi* SBR5, the technology could be adapted to genome editing using the intact Cas9 protein. In the related *Paenibacillus polymyxa* DSM 365, CRISPR-Cas9-based genome editing was developed and used to improve its exopolysaccharide production (Rütering et al. 2017). Our study is the first demonstration of CRISPRi-mediated gene repression in Paenibacilli. Thus, we developed a new tool for genetic manipulation in Paenibacilli such as the plant growth promoter *P. sonchi* SBR5, enabling its study as a strong candidate for crops inoculation in the future.
